# Management of Fifth Metacarpal Neck Fracture (Boxer's Fracture): A Literature Review

**DOI:** 10.7759/cureus.9442

**Published:** 2020-07-28

**Authors:** Malik Hatim Hussain, Ali Ghaffar, Qaisar Choudry, Zafar Iqbal, Muhammad Noman Khan

**Affiliations:** 1 Orthopaedics and Trauma, East Lancashire NHS Hospitals, Blackburn, GBR; 2 Emergency Medicine, California Institute of Behavioural Neurosciences and Psychology, Fairfield, USA; 3 Emergency Department, The Kidney Center, Karachi, PAK; 4 Emergency Medicine, Usman Memorial Hospital, Karachi, PAK

**Keywords:** boxer's fracture, fifth metacarpal fracture, metacarpal fractures, hand injury

## Abstract

Boxer’s fracture is the fifth metacarpal neck fracture resulting from direct trauma to the clenched fist. Worldwide, this type of fracture is the most typical presentation to emergency departments. The management of fifth metacarpal fractures varies from one setting to another. Conservative management is the preferred option for closed, non-angulated, non-malrotated fractures while open fractures, significant angulation, rotational deformity, and intra-articular extension are recognised indications for surgical intervention. The scope of this article covers the results of a literature review examining the management strategies for such fractures.

## Introduction and background

A considerable 33% of patients with hand fractures are metacarpal fractures [1]. A majority of metacarpal fractures involve the fifth metacarpal [2]. A sub-capital/neck of the fifth metacarpal fracture is commonly known as a boxer's fracture [3]. It is more common in males than in females, and incidence peaks at the age group of 10-29 years [4-5]. Usually, these fractures can be managed conservatively, however, several factors like longitudinal shortening, angulation, malrotation, bone loss, and soft tissue injury are indications for surgical fixation [6].

This literature review aims to discuss all possible management options for fifth metacarpal bone fractures.

## Review

Pathoanatomy and diagnosis

**Figure 1:**
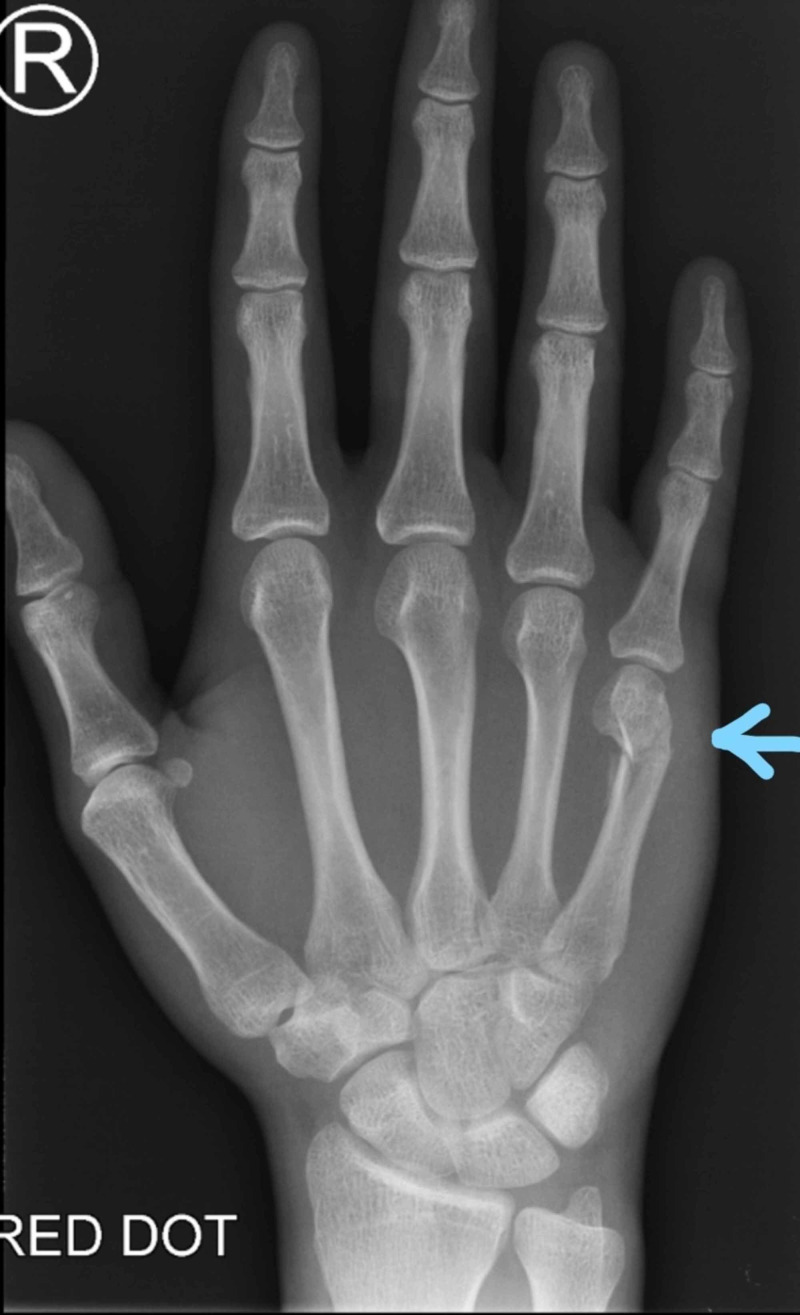
X-ray (PA view) of the right hand shows an extra-articular, comminuted fracture of the neck of the fifth metacarpal PA = Posteroanterior

**Figure 2:**
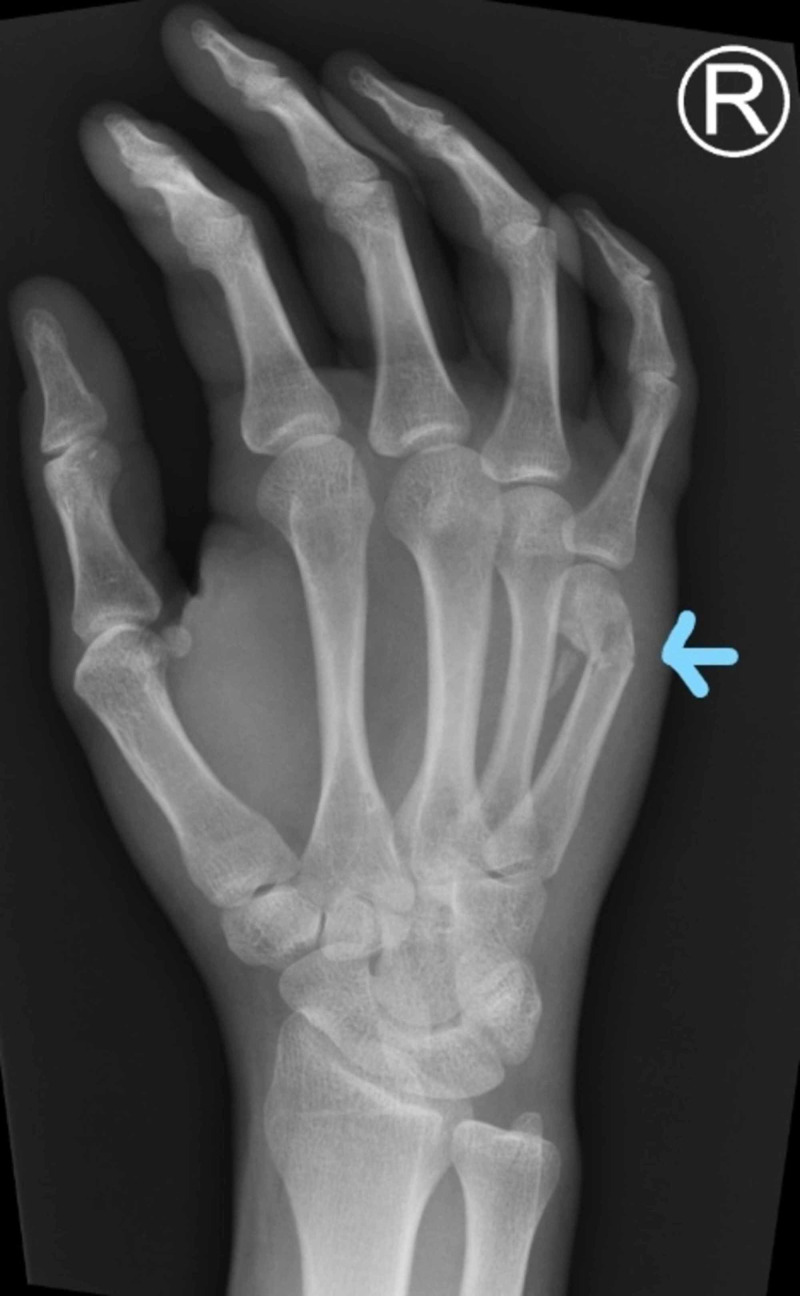
X-ray (oblique view) shows an extra-articular, comminuted fracture of the neck of the fifth metacarpal with volar angulation

A boxer's fracture is typically a result of direct trauma to a clenched fist where energy is transferred through the fifth metacarpal axially and mostly results in apex dorsal angulation due to the pull of the interosseous muscles of the hand [7]. Like any other long bone fracture, metacarpal bone fractures also follow the same descriptive classification pattern, i.e., open or closed, intraarticular or extra-articular, oblique, spiral, transverse, or comminute [8]. The neurovascular bundle runs adjacent to a metacarpal and may become damaged in displaced fractures, which requires surgical intervention [7]. While examining a potential fifth metacarpal fracture, a clinician must give special attention to check for any breaks in the skin (fight bites), neurovascular status, pseudo clawing, or rotational alignment, and the uninjured hand should be compared [7]. Early thorough debridement and antibiotics are required for patients with fight bites, as amputation might be required in certain cases with chronic bone and tendon sheath infection [9]. Plain X-rays (anteroposterior, lateral, and oblique) are the gold standard for diagnosis and for determining angulation (Figures 1-2). Angulation should be measured at more than 15 degrees, as the normal angulation of the fifth metacarpal neck is 15 degrees [7]. Kocaoğlu S and colleagues recently suggested the use of ultrasonography for detecting metacarpal fractures in the emergency department [10]. Occult metacarpal fractures require an early computed tomography (CT) scan for prompt diagnosis [11].

Non-operative management

An uncomplicated - closed, not angulated, and not malrotated or otherwise displaced - fifth metacarpal fracture can be managed with initial immobilisation with an ulnar gutter splint [7]. Alternatively, a more minimalistic approach of strapping the little and ring finger together, also known as buddy taping, can be used in uncomplicated cases [12]. Conventional teachings suggest that the position of the hand for fracture splinting should be: the wrist extended at 20 degrees, 60-70 degree of flexion at the metacarpophalangeal (MCP), and interphalangeal joints in extension [13]. The risk of rotational deformity is reduced significantly by buddy strapping/taping [14]. Complete immobilisation with an ulnar gutter or buddy strapping, which provides immediate motion have relatively comparable functional outcomes [8,15]. However, a study conducted by Bansal R reported a higher degree of patients satisfaction, less follow-up, and an earlier return to work when treated with buddy strapping comparatively to splinting [15]. Many authors recommend accepting angulation up to 70 degrees in small finger metacarpal neck fractures [16-18]. Despite these recommendations, a biomechanical study conducted in 1999 concluded an 8% loss in flexor digiti minimi grip strength and a 22% reduction in the range of motion associated with 30 degrees of angulation, therefore, suggests an upper limit for acceptable angulation of 30 degrees [19]. Boxer's fracture with a significant fracture requires closed reduction [7]. Closed reduction of angulated fractures can be attempted in an Accident and Emergency department (A & E) setting; 90-degree flexion is attained at the little finger metacarpophalangeal and interphalangeal joints. Pressure is then applied to the dorsal aspect of the fracture to push the head dorsally flattening the shaft [20]. Although this technique is successful in reducing fractures, the maintenance of reduction remains a vexing problem [14]. Harris and colleagues presented a paper describing the longitudinal traction of the fifth metacarpal with subsequent immobilisation with a cast. Results indicated over 80% of initial correction of angulation with only 1 degree of loss of correction after discontinuation of the cast at three to four weeks [21].

Operative management

There is a considerable amount of variability in the preferred method for surgical fracture management of the fifth metacarpal fracture also known as the Boxer’s fracture. We reviewed the available literature in an attempt to summarise the methods used in the current practice to reach a consensus. Two previous studies, including a meta-analysis, showed comparable levels of subjective satisfaction with both conservative and surgical management. However, objectively, the operative groups showed lower levels of residual dorsal angulation, albeit, with longer rehabilitation periods and time out of work relative to the conservatively managed group of patients. Both the conservative and operative groups exhibited some degree of rotational mal-alignment. However, neither of the two methods resulted in any significant loss in the range of movement or grip strength [22-23]. With the above considered, surgical management should only be preferred when there is a strong indication for it. The following are some of the indications for surgical management that have been mentioned in the literature [24-27].

1. Open fractures: prompting irrigation and debridement of the wound, along with open reduction and internal fixation;

2: Compound fracture or multiple fractures involving multiple metacarpals and/or phalanges;

3: Intra-articular fractures particularly when a fragment prevents the smooth motion of the joint;

4: Fractures extending into the head of the metacarpal with >1 mm displacement;

5: Volar angulation and displacement of the distal fragment. This is a rather unique criterion in terms of its relevance to which metacarpal is involved. Volar angulation can result in reduced grip strength, pseudo clawing, and a visible deformity of the metacarpal head on the palmar aspect. Moreover, ulnar metacarpals have greater compensatory metacarpocarpal joint flexibility and hence can tolerate a greater degree of volar angulation when compared to the radial metacarpals. Acceptable volar angulations of the distal fragment are 10, 20, 30, and 40 for the second, third, fourth, and fifth metacarpals, respectively [25];

6: Shortening of >5 mm;

7: Malunion or non-union;

8: Inability to reduce a fracture with conservative methods; and

9: Rotational deformity.

Fixation technique

Different surgical techniques are currently in use for the surgical management of the boxer’s fracture. The ultimate decision as to which should be used depends on the surgeon's preference when taking into account both the pros and cons of each technique and the pathoanatomy of the individual case.

Kirschner Wires (K-Wires)

Most closed simple metacarpal neck fractures can be managed conservatively by flexing the digit at the metacarpophalangeal and proximal interphalangeal joints and applying a dorsally directed force along the plane of the first phalanx. Stable fractures can then be splinted externally. However, if the fracture is deemed to be unstable then further stabilisation by pinning with K-wires can be employed [25]. K-wires are minimally invasive and easy to use as an implant for both percutaneous and open fracture stabilisation. However, there are certain cons with using K-wires such as possible neurovascular injury, tendon adhesions, pin site infection, and pin loosening [24-25]. Available in different diameters, K-wires are driven using a drill into the bone in the ulnoradial, radioulnar direction or through the centre of the bone. The K-wire should pass the fracture site ideally at an angle of greater than 45 degrees [25]. K-wires are inserted using four techniques (cross-pinning, crucifix pinning, bouquet pinning, and single K-wire in lazy S fashion), which are described below [24,27-28].

Cross-pinning:* *Two wires are inserted bicortically in a retrograde fashion from a point of entry distal to the fracture site. The pins are prevented from crossing at the fracture site to avoid rotational changes in the fragment. A 0.9 or 1.1 mm diameter K-wire is used.

Crucifix pinning: A thicker 1.6 mm diameter wire is advanced through the head of the metacarpal retrogradely into the medullary canal. The second thinner wire is driven from the radial aspect into the heads of the fractured metacarpal and an adjacent metacarpal, forming a crucifix shape.

Bouquet pinning: Multiple, typically three, K-wires are driven anterogradely centrally through the intramedullary cavity. The resulting shape of the wires resembles a bouquet, as the distal ends are typically given a dorsal bend to support the reduction. 

Single K-wire in ‘lazy-S’ fashion: A study has reported encouraging results with only one out of 28 patients requiring repeat fracture fixation at the final follow-up. A single K-wire is given a mild bend at the 5-mm point, with an opposing but much smoother curve further along the K-wire. The wire is driven antegradely into the medullary canal. The study reported that at the final follow-up, there was no evidence of rotational or angulation deformity [29].

Transverse pinning*: *Typically used for 4th and 5th metacarpal fractures, K-wires are driven from the ulnar aspect into the fifth and fourth metacarpal to stabilise the fracture fragment to the unfractured adjacent counterpart. Two studies compared the results of this technique to intramedullary pinning and discovered that operative times were shorter and the rate of complications was lower for transverse pinning. However, the intramedullary technique produced better functional results [27-28].

Intramedullary Fixation

As discussed above, a single K-wire can also be used for intramedullary fixation. However, two other methods have been reported in the literature for intramedullary fixation of the fractured fragment; prefabricated commercially available nails and headless screws. Generally, the nails are placed anterogradely and screws are placed retrogradely [24]. When compared to K-wire cross pinning, intramedullary fixation was shown to produce an improved range of motion and lower incidences of shortening [24,30]. A study retrospectively followed the outcomes with the usage of headless intramedullary screws for metacarpal neck and shaft fractures and concluded that the functional outcomes were excellent, producing a total range of motion of more than 240 degrees [26]. However, Padegimas et al. support the use of headless screws for neck fractures only [24]. A headless screw is inserted through a small incision at the metacarpophalangeal joint along a guide-wire drilled retrogradely into the distal fragment. The screw is buried into the bone, which precludes the need for subsequent removal. Tobert et al. suggested that this characteristic, along with improved rotational stability of the fracture reduction, offers an advantage over other techniques, such as K-wires, which require a repeat procedure to remove the wire [26]. Since the MCP joint surface is implicated during the insertion of the screw, violation of the articular surface is a concern to bear in mind. However, the study does not report any long-term consequences [26]. Headless intramedullary screws have been reported to produce similar biomechanical stability as compared to Kirschner wires [26].

Plate and Screw Fixation

The plate and screw construct is traditionally favoured for its superior biomechanical stability as compared to other methods [24]. However, a focused study found no significant differences in the peak load and stiffness profile of the bone for fixation with plate and screw versus K-wires. The study, however, only focused on the CMC joint fixation using cadaveric bones [31]. Other potential benefits of the plate and screw fixation include ease of fixation when there is significant comminution is present or there are multiple metacarpal neck fractures [24]. However, a small distal fragment can reduce distal purchase for screw fixation, making this method unusable [26]. The method has also been reported to produce significant stiffness and extensor mechanism complications [24]. Facca et al. compared the benefits of using a locking plate versus K-wires and discovered that although locking plates offer an obvious advantage of immediate mobilisation as compared to K-wires, which require six weeks of immobilisation, the loss of mobility and higher cost of the procedure did not justify the usage [32].

## Conclusions

Uncomplicated fifth metacarpal fractures are usually treated with either immobilisation and splinting or neighbour strapping, with a comparable degree of functional results. Fractures with significant angulation require closed reduction and application of splint. The acceptable degree of angulation is debatable among authors, with the majority accepting angulation of up to 70 degrees. We found that there are very specific indications for surgical management to be preferred over the conservative approach. Furthermore, morphological variations in the nature of the fracture favour certain surgical fixation methods over the other. Despite these, there is no consensus on the superiority of any single surgical technique.
